# Developing reference plans for evaluating global clinical trials credentialing and PSQA systems

**DOI:** 10.1002/acm2.70113

**Published:** 2025-05-29

**Authors:** Fre'Etta M. D. Brooks, Mohammad Hussein, Jessica Lye, Christopher L. Nelson, Nakamura Mitsuhiro, Mallory C. Glenn, Patricia Diez, Rushil Patel, Maddison Shaw, Ileana Silvestre Patallo, Miriam Barry, Catharine H. Clark, Joerg Lehmann, Stephen F. Kry

**Affiliations:** ^1^ University of Washington Medical Center Seattle Washington USA; ^2^ Metrology for Medical Physics National Physical Laboratory Teddington UK; ^3^ Olivia Newton John Cancer Wellness and Research Centre, Austin Health Melbourne Australia; ^4^ Australian Clinical Dosimetry Service, ARPANSA Melbourne Australia; ^5^ RMIT University Melbourne Australia; ^6^ Department of Radiation Physics University of Texas MD Anderson Cancer Center Houston Texas USA; ^7^ Department of Advanced Medical Physics Graduate School of Medicine Kyoto University Kyoto Japan; ^8^ Imaging Radiation Oncology Core Houston Texas USA; ^9^ Radiotherapy Trials Quality Assurance Group Northwood UK; ^10^ Australian Radiation Protection and Nuclear Safety Agency Melbourne Australia; ^11^ Department of Radiotherapy Physics University College London Hospital London London UK; ^12^ Department of Medical Physics and Bioengineering University College London London UK; ^13^ School of Information and Physical Sciences University of Newcastle Newcastle Australia; ^14^ Department of Radiation Oncology Calvary Mater Hospital Newcastle Australia; ^15^ Institute of Medical Physics University of Sydney Sydney Australia

**Keywords:** internation dosimetry audit, IMRT/VMAT, sensitivity specificity evaluation, quality assurance, dosimetry audit comparison

## Abstract

**Purpose:**

To develop a practical framework for creating a diverse set of validated reference plans (varying in complexity) and implement a workflow to introduce beam modeling, calibration, and delivery errors into the reference cohort to test and compare various dosimetry audit methodologies.

**Methods:**

Sixteen IMRT and VMAT reference plans were created, using RayStation software, for four phantom geometries based on established credentialing cases from participating Global Harmonization Group (GHG) members. These reference plans were first validated in a multi‐ion‐chamber phantom. Nine dosimetric errors (perturbations) were introduced into the plans by modifying beam model and/or delivery parameters (MLC‐offset, MLC‐transmission, leaf‐tip‐width, PDD, beam calibration, and MLC‐position) based on documented community distributions of errors; this produced 144 plans. The dose impact on the clinical target volume (CTV) and organs at risk (OARs) was determined, and a range of classifications was developed to determine if the perturbed plan should pass or should fail an audit.

**Results:**

Introducing errors into the reference plans impacted each plan differently. Dose perturbations ranged from <1% to >10% in the mean dose to the CTV and <10% to >30% in the near maximum dose to OAR (D0.03). The 144 plans included clear “acceptable” and “unacceptable” scenarios, with significant changes in dose (relative to baseline reference values), as well as near pass/fail threshold results. Plan complexity was found to have a strong impact on dose deviation, and the mean MLC Gap metric was found to best capture this relationship.

**Conclusion:**

This study presented a framework to develop a set of reference plans and perturbations that can be used to assess and compare various audit and PSQA methodologies. The GHG has developed this framework as part of our ongoing work to test the comparability of their audit systems; this framework supports our work of aligning international dosimetry audits across the globe.

## 1 INTRODUCTION

There are substantial inconsistencies in the performance of quality assurance (QA) systems in determining the acceptability of IMRT/VMAT dose delivery accuracy. Despite developing guidance on conducting patient specific quality assurance (PSQA), these guidelines are not well adhered to, and these systems tend to poorly detect unacceptable plans.^1^, ^2^, ^3^, ^4^, ^5^

The inconsistent performance in detecting treatment delivery errors is also of concern in the context of clinical trial credentialing. Different clinical trial QA bodies (such as IROC, RTTQA, TROG, EORTC, and JCOG, e.g.), conduct different credentialing tests to ensure that institutions are able to deliver radiotherapy doses consistently and accurately. These differences in dosimetry audit methodology among various international radiotherapy clinical trial QA agencies can be attributed to a number of historical, logistical and financial reasons.^6^, ^7^, ^8^ To conduct a performance assessment that compares the results among these different auditing bodies, the sensitivity and specificity of the different audit systems to dosimetric errors needs to be understood. It is necessary to begin with a framework that allows for adequate selection of reference and perturbed plans for the assessment of system sensitivity and specificity. This process requires a cohort of suitable (error‐free and accurately calculated by the treatment planning system [TPS]) reference plans, such that the calculated and measured doses agree. Next, clinically relevant perturbations, such as changes to the multi‐leaf collimator (MLC) transmission and leaf gap, and combinations of these, need to be introduced to the cohort. The perturbations should span a range of errors from minor (acceptable) to clinically compromising target coverage or organs at risk (OAR) sparing (unacceptable). After the cohort of plans is established, each reference plan can be evaluated against plans containing errors by each QA system to assess how accurately each system can classify acceptable or unacceptable plans, thus quantifying the system's ability to detect poor beam models or delivery issues.

The work in this study aligns with the framework under development by AAPM/ESTRO TG‐360 that seeks to address the challenges associated with IMRT plan evaluation. The underlying philosophy of TG‐360 is that audit and PSQA systems should be evaluated against a cohort consisting of plans that are both acceptable and unacceptable to determine if/when the systems correctly pass or fail the plans.^9^

The Global Quality Assurance of Radiation Therapy Clinical Trials Harmonization Group (GHG) is a consortium comprised of organizations around the world that perform clinical trial QA. A main objective of the GHG is to harmonize and improve the QA of radiotherapy implemented worldwide as it pertains to multi‐institutional cooperative clinical trials for the treatment of cancer.^10^ For example, such harmonization would allow successful credentialing from one group to be accepted by other credentialing groups, and vice versa. The establishment of such reciprocity would serve to combine the efforts amongst participating institutions, thereby increasing the number of clinical trials that can impact the radiotherapy community.^11^, ^12^, ^13^, ^14^, ^15^, ^16^

This study focuses on establishing a practical framework for creating a set of validated reference plans and a workflow for developing plans that could be used to test and compare the differentiability of various audit methodologies in a manner that has not been previously explored. The work shown represents the first steps necessary to evaluate the sensitivity and specificity of clinical trial auditing systems and is also relevant for clinical PSQA systems where similar issues exist. This study aims to provide both a procedural foundation for audit methodology and PSQA evaluation as well as insight into the magnitude of perturbation associated with beam modeling, calibration, and delivery errors, including the interplay of plan complexity on these perturbations.

## 2 METHODS

### 2.1 Reference plan development

Six international GHG members participated in developing the proposed IMRT dose assessment framework: the Australian Clinical Dosimetry Service (ACDS) [Australia], the Imaging Radiation Oncology Core (IROC) [United States of America], the Japan Clinical Oncology Group (JCOG) [Japan], the National Physical Laboratory (NPL) [United Kingdom], the National Radiotherapy Trials Quality Assurance Group (RTTQA) [United Kingdom], and the Trans‐Tasman Radiation Oncology Group (TROG) [Australia].

**FIGURE 1:**
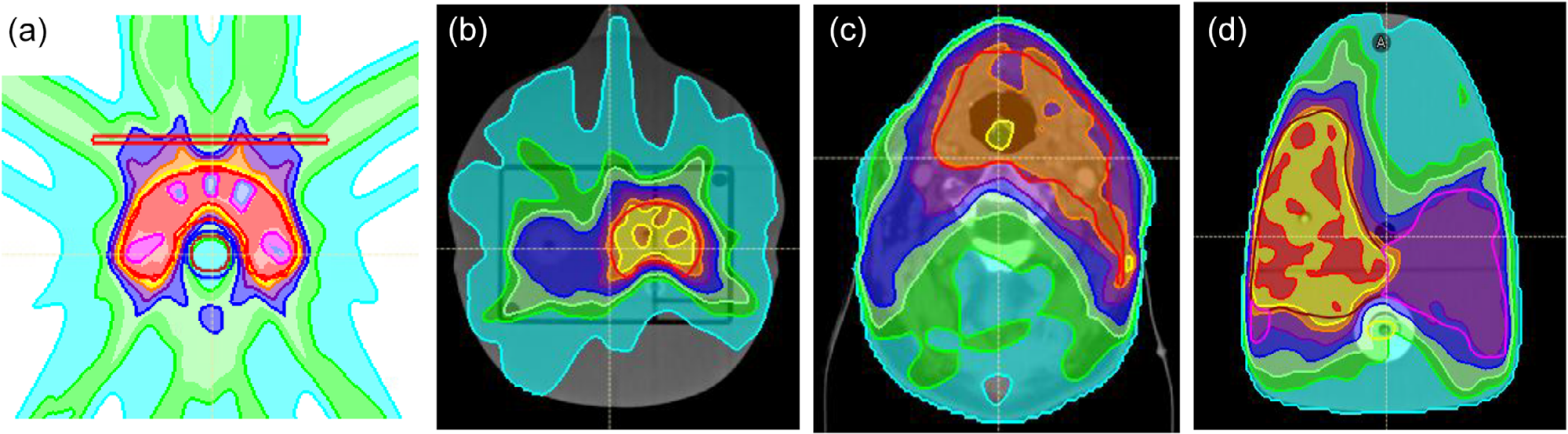
Planning cases in the original planning geometry: (a) ACDS used a credentialing phantom from TG119 report, C‐shaped target. The remaining methodologies are based on Head and Neck geometry, as one of the most complex anatomical scenarios: (b) IROC, (c) RTTQA, and (d) NPL. ACDS, Australian Clinical Dosimetry Service; IROC, Imaging Radiation Oncology Core; NPL, National Physical Laboratory; RTTQA, National Radiotherapy Trials Quality Assurance Group.

Four different geometries were used, (Figure 1), which represent clinical trial audit geometries. The four geometries are based on H&N‐type anatomies and are all used generically by different clinical trial QA groups. Two IMRT and two VMAT plans were created for each of the four geometries (Figure 1), with varying levels of plan complexity; yielding a total of 16 plans. These audit reference plans were designed using the RayStation TPS [RaySearch Laboratories] for an Elekta VersaHD machine using six MV beams. The RayStation beam model was commissioned using the median radiotherapy community TPS data for RayStation, as defined in Glenn et al. (2020), and median linac‐specific reference data for the VersaHD, as defined in Kerns et al. (2016). These median values have been previously shown to be strong representations of typical radiotherapy equipment.^17^, ^18^

### 2.2 Validation of reference plans

To detect errors in plans, it is necessary that the original plan (without errors) be accurately calculated by the TPS. If the original plan is calculated incorrectly, biases in the dose agreement will disrupt the apparent sensitivity and specificity of the system (e.g., if a +5% error is introduced into a plan that has a systematic −5% dose offset, the error would show perfect agreement). Reference plans were first validated to confirm that each plan was error‐free (within a defined tolerance margin), correctly calculated by the TPS, and therefore suitable for use as a reference cohort. This served to provide a baseline from which any introduced perturbation impact could be quantified and later utilized to assess individual system differentiability (sensitivity and specificity).

**FIGURE 2:**
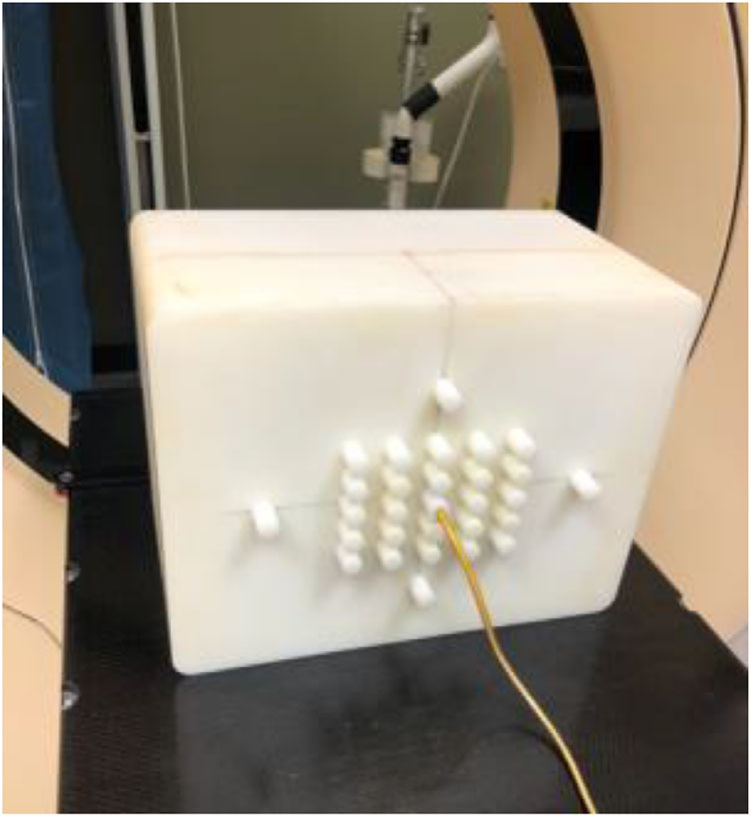
Multi‐ion chamber validation phantom.

To create an independent measurement‐based validation, a multi‐ion chamber phantom consisting of a cube made of high‐impact polyethylene with A1SL chambers (Exradin 0.057cc) [Standard Imaging] was used, shown in Figure 2. The plans were transferred to the validation phantom and the dose distribution for each plan was aligned such that numerous ion chamber locations were in the high dose region. The measurements were then repeated for each plan, using a different isocenter, creating a total of eight distinct measurement locations in the validation phantom. Point dose differences were assessed between the mean calculated dose over the contour of the ion chamber's active volume and the measurement (local percent difference). Measurement points in a high gradient (>10% across the measurement location) or low dose region (<50% relative to the maximum dose) were excluded. Finally, any measurement locations demonstrating differences between the delivered and expected dose, exceeding 4% with an average absolute agreement of 2% among all points, were excluded from the reference cohort. Plan acceptability (to be “reference plans”) was established based on the results found in Table 2, balancing the need to exclude plans that would introduce bias against the practical need to establish realistic criteria. Any plans that failed to meet the criteria were rejected and replanned.

### 2.3 Introduction of errors

Next, a set of error modes was developed. The errors were inspired by clinically realistic beam modeling deficiencies, MLC calibration limitations, and beam output calibration errors.^17^, ^18^, ^19^, ^20^, ^21^ In particular, these errors included modifying the MLC offset, transmission, and leaf tip width parameters to match community values used at different percentile levels. It also included machine calibration errors (1% or 2%); a PDD error (based on observed errors in the community); and an MLC calibration error (0.5 mm bank offset). A final “error” was implemented, which was to use an alternate fully commissioned beam model.

These perturbations were specifically selected to be clinically realistic: the TPS parameters (MLC offset, transmission, leaf‐tip width, PDD), beam calibration, and MLC position were modified based on documented community distributions of error.^17^, ^19^, ^20^, ^21^ The values selected for TPS parameter perturbations were modified to match radiotherapy community data at the 2.5, 25, 75, and 97.5 percentile levels and for their known effect on clinical dose.^17^, ^19^

The nine different perturbations (Table 1), applied to each of the 16 cases, generated 144 plans (including reference and those with errors) that were evaluated in the original planning geometry (Figure 1), using dose‐volume histogram (DVH) assessment. The differences in the mean dose to the CTV coverage and the near‐max dose (D_0.03_) to the OAR (spinal cord structure) of each plan, relative to the unperturbed reference plan, were calculated.

**TABLE 1:** The parameter perturbations, calibration, and delivery errors used to create the different error modes to produce plan perturbations.

Plan perturbations	Changed parameter(s) relative to the reference plan
1	MLC(O) 2.5
2	MLC(O) 97.5
3	MLC(O) 25, MLC(T) 25, −1% output offset
4	MLC(O) 75, MLC(T) 75, +1% output offset
5	MLC(T) 2.5, −2% output offset, MLC leaves closed 0.5 mm
6	MLC(T) 97.5, +2% output offset, MLC leaves open 0.5 mm
7	PDD 2.5, MLC(LTW) 2.5, −1% output offset
8	PDD 97.5, MLC(LTW) 97.5, +1% output offset
9	Alternate beam model

*Notes*: The parameter perturbations were based on radiotherapy community data at the 2.5, 25, 75, and 97.5 percentile levels. MLC offset: MLC(O), MLC transmission: MLC(T), MLC leaf tip width: LTW, and percent dose depth: PDD.

Abbreviation: MLC, multi‐leaf collimator.

### 2.4 Complexity assessment

As different plans showed different dosimetric impacts from the same introduced error, there was a clear need to evaluate this quantitatively. The complexity of each plan was therefore assessed to provide insight into the relationship between plan complexity and sensitivity to perturbations due to the introduced errors. Linear regression was used to assess the correlation between the average magnitude of mean dose differences in the CTV and plan complexity scores.

The following complexity metrics were considered: modulation complexity score (MCS), edge metric, mean Tongue and Groove Index (T&Gi), mean MLC Gap, monitor units (MU), and plan irregularity (PI).^7^, ^22^, ^23^, ^24^ These metrics were averaged across all beams in each plan and were then extracted using in‐house software, PlanAnalyzer, developed by a working group of the Catalan Society of Medical Physicists. The software calculates complexity metrics using the data contained in the DICOM plan.^7^, ^24^ The complexity metric that best fitted the data was determined based on the greatest average R‐squared value.^23^, ^25^

## 3 RESULTS

### 3.1 Validation of reference plans

The differences ([measured‐planned]/planned) between the planned and measured doses for the reference plans are listed in Table 2.

**TABLE 2:** The percentage difference (measurement vs. calculated dose) at measurement locations alongside the maximum percentage dose difference and average magnitude tabulated across all measurement locations in the multi‐ion chamber phantom.

Measurement number	1	2	3	4	5	6	7	8	Absolute maximum difference	Average difference
Plan										
1	−1.4	−3.0	0.4	−0.4	0.1	0.1	*	*	3.0	0.9
2	−0.9	−2.8	0.1	−0.4	−1.1	0.8	*	*	2.8	1.0
3	−1.2	−1.2	−0.6	0.6	−0.5	−0.8	*	*	1.2	0.8
4	−0.5	−1.0	0.2	0.6	−0.4	1.0	*	*	1.0	0.6
5	−0.2	−0.2	2.6	−2.3	0.5	2.9	*	*	2.9	1.5
6	−1.4	−0.6	1.4	−1.9	−0.2	1.3	*	*	1.9	1.1
7	−2.1	0.4	0.2	0.1	−2.0	1.4	1.2	0.1	2.1	0.9
8	−2.9	0.5	−0.7	1.8	−1.8	−0.6	2.2	1.8	2.9	1.5
9	−3.9	−2.4	−0.9	−1.8	−1.6	−0.5	*	*	3.9	1.9
10	−3.5	−1.8	0.8	−2.5	−2.0	0.3	*	*	3.5	1.8
11	−1.3	−1.6	−0.7	−1.3	0.2	−1.5	*	*	1.6	1.1
12	−3.6	−0.5	−1.9	−0.1	−2.3	1.8	*	*	3.6	1.7
13	−2.9	−0.4	0.4	−1.3	0.3	0.3	*	*	2.9	0.9
14	−1.3	−0.4	−0.4	−2.0	0.1	3.9	*	*	3.9	1.4
15	−1.2	0.2	1.0	0.5	2.3	2.6	*	*	2.6	1.3
16	−1.2	−0.2	0.5	−0.5	0.4	1.6	*	*	1.6	0.7
17	−5.7	−4.5	1.0	−4.7	−4.8	5.2	*	*	5.7	4.3
18	1.3	4.3	−1.2	3.8	1.0	6.0	*	*	6.0	2.9
19	3.9	−0.1	4.9	−2.4	9.8	−1.4	*	*	9.8	3.8
20	4.3	−7.3	−6.0	−0.2	−2.1	−8.2	*	*	8.2	4.7

*Note*: * denotes measurements that did not meet the high dose/low gradient requirements.

The criteria established were that all measurement points were within 4% of the calculated dose and the average (absolute agreement) of all points was within 2%. The last four plans (17–20) did not meet these criteria and were therefore deemed unacceptable to be reference plans and were excluded from further consideration.

Tightening these criteria further had a dramatic impact on the number of plans that were deemed acceptable. For example, if the criteria were tightened to a maximum deviation of 3% and an average absolute deviation of 1% among all points, the number of acceptable plans in our cohort decreased from 20 to only seven plans. Moreover, the condition that dose agreement must match within 4% is also supported by previous studies. In cases where multiple ion chamber measurements were made, in both Kruse et al.^26^ and McKenzie et al.^3^ a 4% max dose difference criterion was used to define an acceptable plan.

### 3.2 Error distribution

Once the reference plans were validated, we determined the impact the introduced errors had on each plan. Changes in dose caused by introducing perturbations to the validated reference plans (calculated in the original planning geometry) ranged from <1% to >10% in the mean dose to the CTV and <10% to >30% in the OAR D_0.03_. The average impact on mean dose to the CTV and D_0.03_ to the OAR is shown in Table 3.

**TABLE 3:** The average impact on the mean dose to the CTV and D_0.03_ to the OAR, in the original planning geometry, across all plans for a given perturbation are shown.

Perturbation		Avg. diff. in mean dose to the CTV (%)	Avg. diff. in OAR D_0.03_ (%)
1	MLC(O) 2.5	−2.1	−4.5
2	MLC(O) 97.5	7.3	17.2
3	MLC(O) 25, MLC(T) 25, −1% output offset	−1.9	−3.1
4	MLC(O) 75, MLC(T) 75, +1% output offset	3.3	6.4
5	MLC(T) 2.5, −2% output offset, MLC leaves closed 0.5 mm	−6.0	−10.1
6	MLC(T) 97.5, +2% output offset, MLC leaves open 0.5 mm	8.4	16.9
7	PDD 2.5, MLC(LTW) 2.5, −1% output offset	−2.3	−3.0
8	PDD 97.5, MLC(LTW) 97.5, +1% output offset	6.1	12.8
9	Alternate beam model	1.0	1.3

Abbreviations: CTV, clinical target volume; LTW, leaf tip width; MLC, multi‐leaf collimator; OAR, organs at risk; PDD, percent dose depth.

The results in Table 3 show that error modes 2 and 6 had the greatest overall average impact on dose, with 5 and 8 also showing substantial differences. These data highlight that clinically realistic perturbations can manifest as dramatically large errors.

**FIGURE 3:**
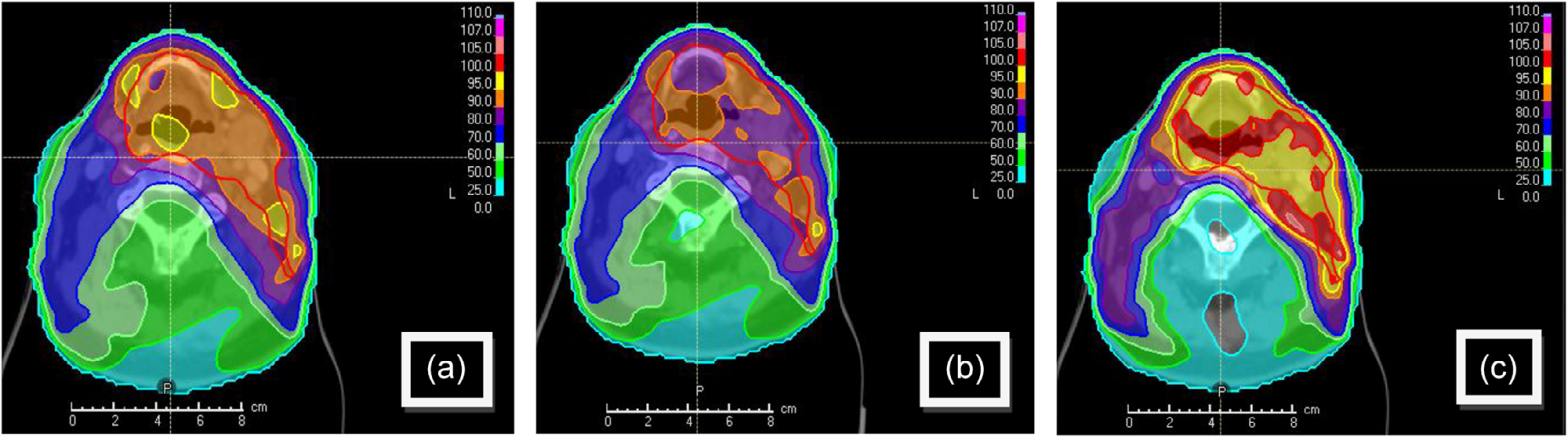
Changes in target coverage in the original planning geometry for an RTTQA case (Figure 1) are shown for the: (a) reference 50‐percentile, (b) error mode 5 (Table 1), demonstrating under‐dosing effects, and (c) error mode 2 demonstrating over‐dosing effects. RTTQA, National Radiotherapy Trials Quality Assurance Group.

Figure 3 illustrates the changes in dose coverage with the introduction of perturbation 5, showing a reduction in the mean dose to the CTV (b) and perturbation 2, showing an increase in the mean dose to the CTV (c).

Each plan was also found to be affected differently by each error mode. The differences in dose averaged across all plans for each error mode are shown in Table 4. The magnitude of the effect on the mean dose to the CTV of the nine perturbations, across all the plans, ranged from 2.4% to 5.8%. The average magnitude of impact on the OAR D_0.03_ ranged from 3.0% to 14.4%. In general, the OAR exhibited twice the variation in magnitude to plan perturbations as observed in the CTVs.

**TABLE 4:** Range and average of the mean dose to the CTV and maximum and average maximum dose to OAR D_0.03_, calculated in the original planning geometry, for all error modes are tabulated below.

Plan number	Range in mean dose to the CTV	Average mean dose to the CTV	Maximum OAR D_0.03_	Average maximum OAR D_0.03_
1	−6.9 to 10.4	5.3	27.5	12.0
2	−6.7 to 9.8	4.9	17.9	8.2
3	−6.4 to 10.9	5.5	30.2	12.1
4	−6.9 to 9.3	4.4	20.3	7.5
5	−7.2 to 11.6	5.1	19.3	8.3
6	−3.2 to 3.9	1.9	5.9	2.9
7	−7.4 to 12.8	5.6	29.4	10.6
8	−6.5 to 9.8	4.3	16.2	6.6
9	−6.7 to 8.4	4.3	31.0	14.4
10	−5.0 to 6.1	3.1	11.8	5.9
11	−7.0 to 12.3	5.8	36.5	14.2
12	−6.6 to 9.2	4.6	17.7	7.9
13	−4.0 to 5.6	2.7	10.2	3.0
14	−3.7 to 4.9	2.4	9.7	3.9
15	−7.5 to 13.1	5.7	33.1	12.2
16	−5.2 to 5.1	3.0	8.4	4.3

Abbreviations: CTV, clinical target volume; OAR, organs at risk.

As the final step, the introduced errors were evaluated to determine if the dosimetric impact would be reasonably interpreted as an “acceptable” or “unacceptable perturbation”. While this decision remains a clinical decision that depends on the patient's condition and treatment intent, PSQA acceptability is overwhelmingly defined based on physics‐defined tolerances. With this in mind, the perturbation in the mean dose to the CTV for each of the 144 plans was used to define the plan acceptability. Three different dose error thresholds in the CTV were considered: a 3% mean difference, a 5% mean difference, and a 7% mean difference. Based on these tolerances, the number of plans (for each error mode) that should pass (“expected to pass”) or should fail (“expected to fail”) an audit is shown in Table 5. The 5% threshold was considered as dose deviations greater than ±5% in the intended dose to the CTV and OAR can adversely affect clinical outcomes.^27^, ^28^ All three of these values are of interest when evaluating the performance of audit systems as there needs to be flexibility on the clinical tolerances (due to local criteria).

**TABLE 5:** Total cohort expected pass/fail distribution based on changes of ±3%, 5%, and 7% to the mean dose to the CTV, calculated in the original planning geometry (Figure 1).

	Mean dose to the CTV (±3%)		Mean dose to the CTV (±5%)		Mean dose to the CTV (±7%)	
Error mode	Expected to pass	Expected to fail	Expected to pass	Expected to fail	Expected to pass	Expected to fail
1	14	2	16	0	16	0
2	3	13	5	11	6	10
3	16	0	16	0	16	0
4	6	10	16	0	16	0
5	0	16	4	12	12	4
6	0	16	4	12	6	10
7	13	3	16	0	16	0
8	0	16	3	13	9	7
9	16	0	16	0	16	0
Total	68	76	96	48	113	31

Abbreviation: CTV, clinical target volume.

### 3.3 Complexity

**FIGURE 4:**
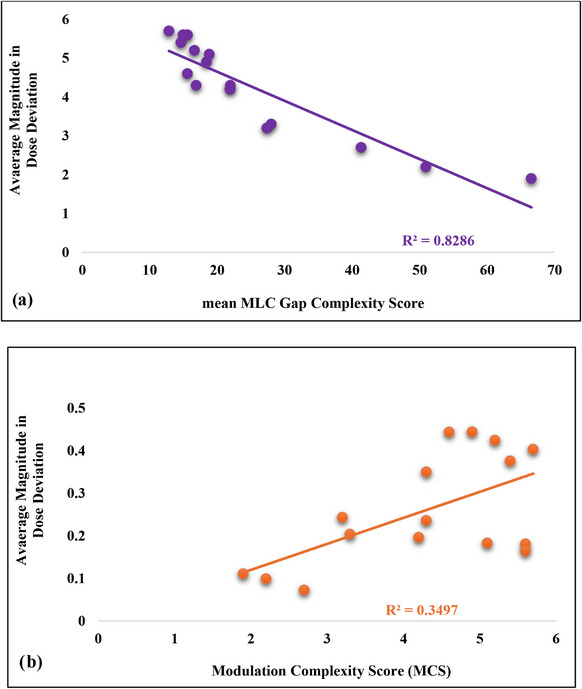
The relationship between two complexity metrics, (a) mean MLC Gap and (b) MCS, and the average magnitude in mean dose deviation to CTV caused by introducing errors into each reference plan. Error modes 2, 5, 6, and 8 (Table 1) were used in the complexity assessment as they demonstrated the greatest impact on dose (Table 3). CTV, clinical target volume; MCS, modulation complexity score; MLC, multi‐leaf collimator.

Plan complexity was utilized as a metric for explaining the different sensitivities of plans to the same error (as shown in Table 4). Figure 4 highlights the relationship between plan complexity and dose deviation (mean dose to the CTV) for two different complexity metrics.

Regression analysis emphasized the differences in predictive power between the two complexity metrics shown in Figure 4. Mean MLC Gap showed a very high correlation between plan complexity and dose error (*R* ^2^ = 0.8286), while MCS showed a much poorer correlation (*R* ^2^ = 0.3497).

**FIGURE 5:**
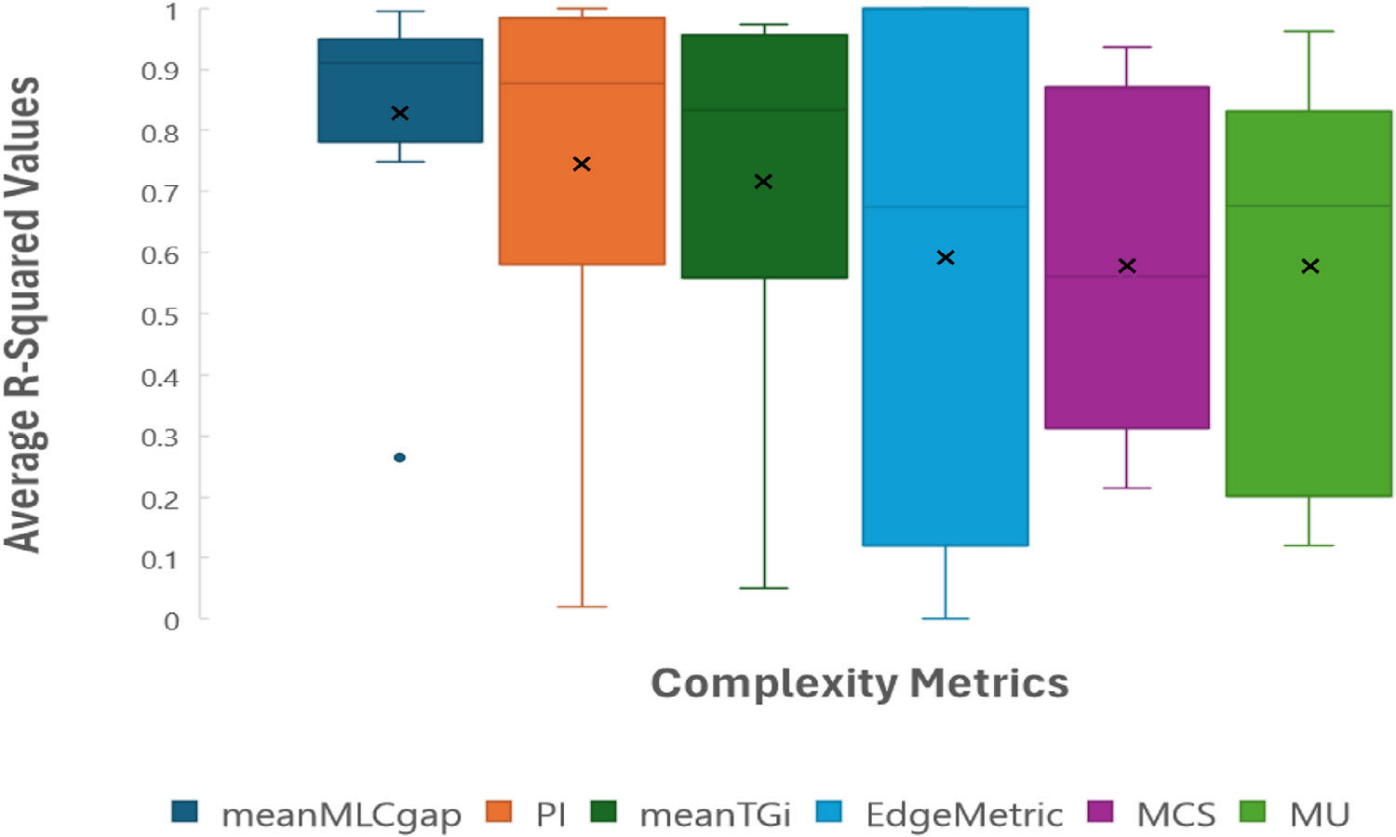
From left to right: The highest to lowest average R‐squared value associated with the regression analysis for each complexity metric: mean MLC Gap, PI, mean T&Gi, Edge metric, MCS, and MU. The average R‐squared values represent the correlation between plan sensitivity to the introduced errors and each of the 6‐complexity metrics evaluated. The “x” inside each box represents the mean value. MCS, modulation complexity score; MLC, multi‐leaf collimator; MU, monitor units; PI, plan irregularity; T&Gi, Tongue and Groove Index.

The relationship between the complexity metric score for each plan and the average magnitude in dose deviation (from the reference values) in the mean dose to the CTV was evaluated using linear regression for error modes 2, 5, 6, and 8 (Table 1). The average R‐squared values were tabulated and compiled in Figure 5. The mean MLC Gap best predicted the impact of plan perturbations on dose calculation.

The plans that demonstrated the greatest sensitivity to error modes were also the plans with the greatest complexity scores, emphasizing the need to include a range of plan complexities when creating a cohort of reference plans to assess different auditing methodologies and PSQA system performance.

## 4 DISCUSSION

The current study presents a methodology to develop a set of reference plans and perturbations of interest for global auditing and PSQA programs. We were able to reinforce previously suggested acceptability criteria for the definition of reference plans, by using 4% (max dose) and 2% (mean dose) deviation values.^3^, ^26^ We were able to introduce clearly unacceptable errors into realistic treatment plans by using clinically documented deficiencies in beam modeling and calibration.

The introduced perturbations were based on deviations observed in clinical practice. In some cases (machine calibration and PDD errors), these represented true errors measured at institutions and mismatch of the TPS versus commissioning PDD data.^29^, ^30^ In other cases (particularly MLC offset or MLC transmission terms in the TPS), the perturbations were based on the range of community practice, spanning numerous machines. It must be considered that not all machines may have the same MLC offset, and therefore these deviations may not correspond to true errors. However, recent data has reinforced that machine values are typically very consistent^31^, ^32^, ^33^, ^34^ and atypical values are significantly correlated with poor audit results, regardless of machine.^35^, ^36^ The MLC leaves being systematically opened/retracted by 0.5 mm was based on a realistic limit of calibration precision, and a value below QA tolerance.^37^ The error modes were selected based on their observance in clinical practice, thus the relevance of these errors adds strength to the study and the foundation created here. It also emphasizes how easily large dose errors may sneak into clinical practice; the need for good quality PSQA is clear, so large errors are always detected. Dose deviations greater than ±5% in the intended dose to the CTV and OAR can negatively impact clinical outcomes.^27^, ^28^ Plan perturbations resulted in dose differences as high as 13.1% and 36.5% in the mean dose to the CTV and maximum dose to the OAR (D_0.03_), respectively. These dose differences greatly exceed the recommended deviation range, and the impact of these findings underscores the importance of careful TPS commissioning and potential delivery error assessment. In general, the OAR demonstrated twice the sensitivity to errors as the CTV, which could merit further evaluation, as gamma is typically assessed in a global setting, thus de‐emphasizing these discrepancies.

We connected the magnitude of dose errors to specific complexity metrics with which the errors were best correlated, providing guidance on what kinds of plans should be included in the reference plan cohort. This approach can be used as the initial step to test and compare the differentiability of various audit methodologies. Future work will include the evaluation of clinical trial QA credentialing systems and patient specific QA devices using this foundation.

Many of the findings of this work support previous findings in the literature, even though the intent of this study is very different. For example, the impact of dose differences due to parameter perturbations (e.g., MLC offset) agrees with prior studies in both RayStation and Eclipse [Varian].^38^, ^39^, ^40^ Further, this study found that mean MLC Gap was the complexity metric that best predicted the impact that plan perturbations have on dose delivery. The same metric, mean MLC Gap, was previously found to be the most correlated complexity metric in a cohort of patient plans (of various anatomies) for which errors in beam were introduced.^19^ This provides further evidence on the importance of selecting the optimal complexity metric, whether that be for preemptively assessing the overall complexity of a test cohort before introducing plan errors, or for evaluating plans in clinical practice.

## 5 CONCLUSION

The current study developed a workflow for designing and validating a cohort of reference plans that meet reasonable and clinically relevant criteria. The introduction of known error modes, based on current radiotherapy community practice, to plans with different complexity, produced a diverse set of plans that included different dose errors. The complexity assessment results also highlight the relationship between plan complexity and plan sensitivity to TPS modeling and calibration; the mean MLC Gap complexity metric best predicted this relationship. Further, the plan perturbations were used to create a classification of plans that should or should not fail an audit; thereby establishing a framework for assessing the differentiability of different audit systems and providing a means for system comparison.

Global collaboration on clinical trials will generate outcomes that are more representative of the diverse population of patients treated worldwide and have a greater impact for the radiotherapy community. Facilitating a high level of collaboration requires the interchangeability of data and agreement among all participating institutions. Acceptable and unacceptable plans should be classified as such, no matter which agencies are auditing or institutions are participating, to ensure consistency in the quality of radiotherapy treatments worldwide. The GHG has developed this framework to test their systems. The proposed framework supports the ongoing work of aligning international dosimetry audits across the globe.

## AUTHOR CONTRIBUTIONS

Fre'Etta M.D. Brooks, Mohammad Hussein, Jessica Lye, Nakamura Mitsuhiro, Patricia Diez, Rushil Patel, Maddison Shaw, Ileana Silvestre Patallo, Miriam Barry, Catharine H. Clark, Joerg Lehman, and Stephen F. Kry conceived the presented work. Fre'Etta M.D. Brooks, Mohammad Hussein, and Mallory C. Glenn created the beam models. Mohammad Hussein, Jessica Lye, Nakamura Mitsuhiro, Patricia Diez, Rushil Patel, Christopher L. Nelson, Catharine H. Clark, Joerg Lehman, and Stephen F. Kry assisted Fre'Etta M.D. Brooks with data collection. Analysis and interpretations were primarily conducted by Fre'Etta M.D. Brooks, Mohammad Hussein, Jessica Lye, Nakamura Mitsuhiro, Patricia Diez, Rushil Patel, Catharine H. Clark, Joerg Lehman, and Stephen F. Kry. The manuscript was drafted primarily by Fre'Etta M.D. Brooks and Stephen F. Kry, however, all authors provided feedback before the final draft submission.

## CONFLICT OF INTEREST STATEMENT

The authors declare no conflicts of interest.
